# Efficacy and safety of SGLT2 inhibitors in acute heart failure: a systematic review and meta-analysis of randomized controlled trials

**DOI:** 10.3389/fcvm.2025.1543153

**Published:** 2025-05-01

**Authors:** Ali Ibrahim Rahil, Tirth Bhavsar, Romman Fatima, Aparajitha Rajkumar, Joy Kumar, Hanif Al Majidan, Namrata Gajjala, Wodwentzky Lefranc, Fnu Deeksha, Harshitha Lingegowda, Muhammad Ehsan, Wajeeh Ur Rehman, Hasan Ahmad, Raheel Ahmed

**Affiliations:** ^1^ Department of Medicine, Hamad Medical Corporation, Doha, Qatar; ^2^ Department of Medicine, Smt. NHL Municipal Medical College, Ahmedabad, Gujarat, India; ^3^ Department of Medicine, Shadan Institute of Medical Sciences, Hyderabad, India; ^4^ Department of Medicine, Government Kilpauk Medical College, Chennai, India; ^5^ Department of Medicine, Kasturba Medical College, Manipal, India; ^6^ Department of Medicine, Universitas Padjadjaran, Bandung, Indonesia; ^7^ Department of Medicine, University Notre-Dame of Haiti, P-au-P, Haiti/Medicine, Lakeside Medical Center, Belle-Glade, FL, United States; ^8^ Department of Medicine, Government Medical College, Patiala, India; ^9^ Department of Environmental Health, Johns Hopkins University, Baltimore, MD, United States; ^10^ Department of Medicine, King Edward Medical University, Lahore, Pakistan; ^11^ Department of Medicine, United Health Services, Johnson City, NY, United States; ^12^ National Heart and Lung Institute, Imperial College London, London, United Kingdom

**Keywords:** SGLT2 inhibitors, acute heart failure, de-novo heart failure, systematic review, meta-analysis

## Abstract

**Background:**

Acute heart failure (AHF) is a serious medical condition with considerable morbidity and mortality ranging from 20%–30% within the first month following hospital admission. We aimed to evaluate the efficacy and safety of sodium-glucose cotransporter-2 (SGLT2) inhibitors administered within the first five days of hospitalization for AHF.

**Methods:**

We utilized various electronic resources such as MEDLINE, Embase, and the Cochrane Library to retrieve relevant randomized controlled trials (RCTs). The meta-analysis was performed using Revman, where the risk ratio (RR) and mean difference (MD) with a 95% confidence interval (CI) were used for dichotomous and continuous variablesrespectively.

**Results:**

A total of seven trials were included in this review. SGLT2 inhibitors were associated with decreased all-cause mortality (RR = 0.61, 95% CI = 0.40, 0.95; *P* = 0.03), worsening of HF (RR = 0.59, 95%CI = 0.36, 0.97;*P* = 0.04), and GFR (MD: 1.05, 95% CI = 0.68, 1.43; *P* < 0.00001) compared with the control group. There were no significant differences between the two groups regarding readmission for HF, cardiovascular mortality, AKI, hypoglycemia, hypotension, and diuretic efficiency. SGLT2 inhibitors were associated with improved KCCQ-CSS scores (MD: −3.82, 95% CI = −7.51, −0.13; *P* = 0.04).

**Conclusion:**

SGLT2 inhibitors demonstrate overall clinical benefits and a favorable safety profile in acute heart failure, although their impact on readmission rates is limited. Further research is needed to refine patient selection and optimize treatment strategies.

**Systematic Review Registration:**

https://www.crd.york.ac.uk/PROSPERO/view/CRD42024571563, PROSPERO (CRD42024571563).

## Introduction

Heart failure (HF) is a prevalent and serious medical condition (1) and is characterized by significant morbidity and mortality, with a 5-year mortality rate ranging from 50%–75% (1). In addition to its detrimental impact on the quality of life, HF imposes a substantial economic burden, with costs in the United States estimated at $ 30.7 billion annually (2, 3). The prevalence of HF is increasing owing to enhanced life expectancy and improved therapeutic strategies. However, acute HF (AHF) remains a critical concern, with an annual mortality range of 20%–30% within the first month following hospitalization (4, 5). Furthermore, AHF significantly increases the risk of recurrent hospitalizations, with a 20% higher likelihood of re-hospitalization for decompensated HF (6).

Current treatment options for AHF, including loop diuretics, primarily focus on alleviating the congestion and symptoms. Although loop diuretics remain the cornerstone of AHF management, they have not demonstrated a meaningful reduction in mortality and are associated with adverse renal effects (7). The lack of effective treatments that improve long-term outcomes in AHF highlights the need for novel therapeutic strategies.

Sodium-glucose cotransporter-2 (SGLT2) inhibitors are a class of medications with unique antihyperglycemic effects. They act on the proximal convoluted tubules to inhibit glucose and sodium reabsorption, thereby promoting glucosuria and natriuresis in patients with type 2 diabetes (8). Beyond their role in diabetes management, SGLT2 inhibitors have shown outstanding cardiovascular benefits in clinical trials, significantly reducing the risk of cardiovascular morbidity and mortality (9–14). Notably, these medications have been found to lower the risk of hospitalization (worsening HF) and cardiovascular death, regardless of diabetes status and ejection fraction (EF), with benefits observed in both HF with reduced EF (HFrEF) and preserved EF (HFpEF) (15–18). This beneficial “class effect” has been consistently demonstrated across various SGLT2 inhibitors (9–14). The cardiac benefits of SGLT2 inhibitors are mediated primarily via natriuresis and glycosuria. They lead to several other downstream effects, such as improvement in blood pressure and reduction of oxidative stress and inflammation (19).

However, the role of SGLT2 inhibitors in AHF, particularly during the early phase of hospitalization, remains unclear. Previous studies have not adequately explored the initiation of these agents during the first days of AHF admission, and there has been considerable variability in the timing of SGLT2 inhibitor initiation. Moreover, recently published RCTs have highlighted the need for more updated reviews.

Our review aimed to fill this gap by systematically evaluating the efficacy and safety of SGLT2 inhibitors administered within the first five days of hospitalization for AHF. This meta-analysis provides crucial insights into the potential of SGLT2 inhibitors to improve outcomes in patients with AHF.

## Materials and methods

This systematic review and meta-analysis was designed following the recommendations of the Cochrane Handbook for Systematic Reviews of Interventions (20) and was reported according to the Preferred Reporting Items for Systematic Reviews and Meta-Analysis (PRISMA) statement (21). It was registered with PROSPERO with identifier number CRD42024571563. Ethical approval was not required for this study.

### Eligibility criteria

The inclusion criteria were as follows: (1) study design: randomized controlled trials (RCTs) only; (2) patient population: patients presenting within 5 days of being admitted to the hospital with AHF; (3) intervention: SGLT2 inhibitors; (4) control: placebo or standard treatment; and (5) outcome: reporting at least one outcome of interest.

HF, whether as an acute decompensation of existing HF or as newly diagnosed (*de novo*) HF, and both HFrEF and HFpEF, were included regardless of diabetes status.

We excluded studies that: (1) recruited patients with AHF who were administered SGLT2 inhibitors after 5 days or discharged from the hospital; (2) were not RCTs i.e., observational studies (case series, cohorts, case-control studies) and reviews; and (3) were conducted in animal populations.

### Information sources and search strategy

The following electronic databases and trial registers were searched for eligible studies from inception until May 15, 2024: Cochrane Central Register of Controlled Trials (CENTRAL, via The Cochrane Library), MEDLINE (via PubMed), Embase (via Ovid), and Clinical Trials.gov. We also utilized grey literature sources (ProQuest Dissertations and Theses Global, PQDT), reference lists of included studies, and related systematic reviews to retrieve all eligible studies. A combination of relevant keywords and MeSH terms such as “SGLT2”, “sodium-glucose cotransporter-2 inhibitors”, “SGLT2 inhibitors”, “de-novo heart failure”, “decompensated heart failure”, “acute heart failure” were used in multiple combinations in our search strategy. The literature search had no language restrictions.

### Selection process

The articles retrieved through our literature search were uploaded to Rayyan, a software used for screening and retrieval of articles. Following deduplication, two reviewers independently screened the articles based on their titles and abstracts. The remaining articles were subjected to full-text screening based on the inclusion criteria. Any discordance between the two reviewers was resolved by a third reviewer. The selection process is illustrated using a PRISMA flowchart.

### Data collection process and data items

Two authors independently extracted data from the included studies using a structured Excel spreadsheet. The data items included study characteristics (trial design, author name, year of publication, trial name, sample size, and follow-up duration), patient characteristics (age, sex, details of comorbidities, baseline LVEF, and *de novo* HF), intervention and comparator details (dose, duration, and type of drug), and outcome measures. All-cause mortality and readmission for HF were the primary outcomes.

The secondary outcomes included cardiovascular mortality, worsening HF, Kansas City Cardiomyopathy Questionnaire Total Symptom Score (KCCQ-TSS) improvement, diuretic efficiency, glomerular filtration rate (GFR), acute kidney injury (AKI), urinary tract infection (UTI), hypoglycemia and hypotension. AKI was defined as an increase in blood creatinine level of 0.3 mg/dl (26.4 µmol/L) or more within 48 h. Diuretic efficiency was defined as weight change per 40 mg i/v furosemide.

### Risk of bias assessment

The revised Cochrane Risk of Bias tool (RoB 2.0) was used to evaluate the risk of bias based on five potential items (2): (i) bias resulting from the process of randomization, (ii) bias secondary to deviation from the intended intervention, (iii) bias related to missed outcome data, (iv) bias in outcome measurement, and (v) bias due to the selective reporting of results. Two reviewers independently applied the RoB 2.0 tool and a third reviewer resolved any discrepancy between the two reviewers. The risk of bias was categorized as “low”, “high” or “some concern”.

### Statistical analysis

Analyses were performed using Review Manager (RevMan version 5.4, Cochrane Collaboration, 2020). A random effects model was applied using the DerSimonian-Laird variance estimator. The risk ratio (RR) and mean difference (MD) with a 95% confidence interval (CI) were used for dichotomous and continuous data, respectively. Forest plots were used to present the results for each outcome.

We planned to construct a funnel plot to assess publication bias if the number of studies was greater than 10. The chi-square (*χ*^2^) test was used to detect heterogeneity and the *I*^2^ statistics was employed to assess its magnitude. *I*^2^ interpretation was performed according to the Cochrane Handbook for Systematic Reviews of Interventions, section 10.10. *P* < 0.10 was considered statistically significant for the *χ*^2^ test. A subgroup analysis of the primary outcomes was performed based on the type of SGLT2 inhibitor used in the trials. We also performed a subgroup analysis of all-cause mortality based on the follow-up duration of the RCTs.

## Results

### Study selection

**Figure 1:**
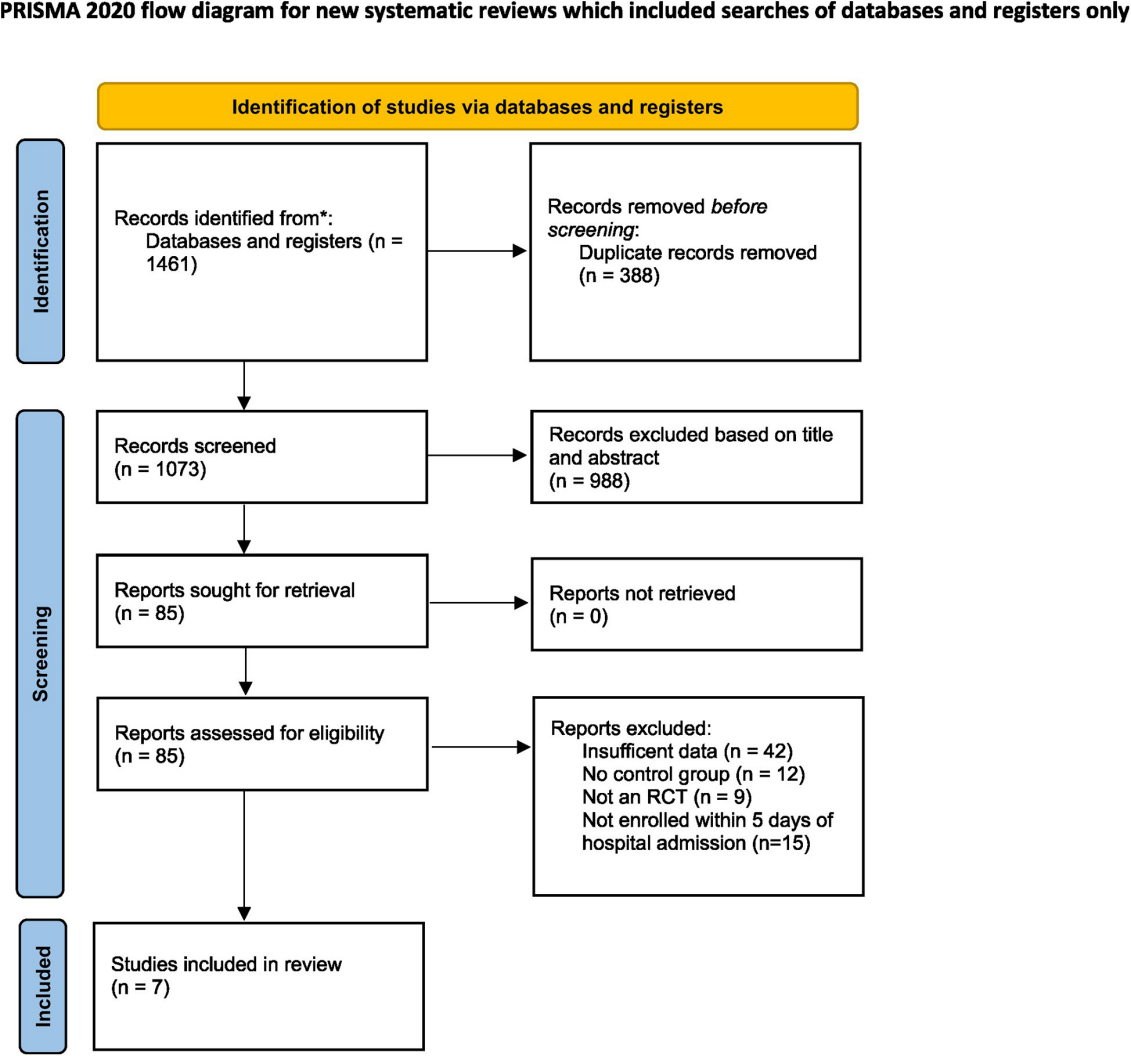
PRISMA flowchart of study selection process.

A total of 1,461 studies were retrieved from various databases and registers. Following deduplication and screening, seven RCTs were included in this meta-analysis (22–28). The detailed selection process is depicted in the PRISMA flowchart (Figure 1).

### Study characteristics

Of the seven included studies (1,181 patients), four evaluated empagliflozin, whereas dapagliflozin was an intervention in the remaining studies. Three studies had standard therapy as a comparator, while the remaining studies used placebo. The time taken between hospital admission and randomization was 24 h in three studies, 12 h in EMPAG-HF, 72 h in two studies, and less than 24 h in one study. Most studies had a follow-up duration of 30 days (57%), with the remaining studies having a follow-up period of more than 30 days. Detailed study and patient characteristics are presented in Table 1.

**Table 1:** Study characteristics of included studies.

Characteristics of included RCTs											
Study ID (First Author, Year)	Location of study	Trial design	No: of study participants (SGLT2i interventional group vs. control)	Intervention (drug and doasge)	Control*	Time between hospital admission and randomization	Follow-up duration	De-novo HF (%)*	Age (years)†	Male (%)	Baseline % LVEF †
Damman et al. (2020) (1)	Netherlands	DB, MC	80 (41 vs. 39)	Empagliflozin 10 mg	Placebo	24 h	60 days	48 vs. 46	79 (73–83) vs. 73 (61–83)	84 vs. 90	36 (19–53) vs. 37 (23–51)
Charaya et al. (2022) (2)	Russia	OP, SC	102 (50 vs. 52)	Dapagliflozin 10 mg	Standard therapy	24 h	30 days	34 vs. 38	72.6 (12.2) vs. 74.2 (11.3)	58 vs. 52	45.6 (15.7) vs. 44.4 (13.6)
Thiele et al. (2022) (3)	NA	DB, SC	19 (10 vs. 9)	Empagliflozin 10 mg	Placebo	3 days	30 days	NA	71.8 (13.4) vs. 72.3 (9.9)	60 vs. 33.3	34 (11) vs. 38 (11)
Schulze et al. (2022) (4)	Germany	DB, SC	60 (30 vs. 30)	Empagliflozin 25 mg	Placebo	12 h	30 days	60 vs. 48	68.8–77.1 vs. 73.4–79.7	63.3 vs. 58.6	45 (39–51) vs. 44 (30–50)
Voor et al. (2022) (5)	Multiple countries	DB, MC	530 (265 vs. 265)	Empagliflozin 10 mg	Placebo	3 days	90 days	33.2 vs. 32.8	70.29 (11.92) vs. 68.94 (14.16)	67.5 vs. 64.9	33.10 (16.39) vs. 34.63 (19.75)
Gilani et al. (2023) (6)	Pakistan	OP, SC	150 (75 vs. 75)	Dapagliflozin 10 mg	Standard therapy	24 h	12 weeks	79 vs. 78	63.76 (10.05) vs. 66.13 (11.73)	82.6 vs. 80	30 (25–30) vs. 30 (20–30)
Cox et al. (2024) (7)	United states	OP, MC	240 (119 vs. 119)	Dapaglifozin 10 mg	Standard therapy	24 h	30 days	14 vs. 13	64.4 (12.7) vs. 64.35 (14.25)	66 vs. 56	42.18 (22.5) vs. 40.3 (26.3)

SC, single-centre; MC, multi-centre; OP, open-label; SB, single-blind; DB, double-blind; NA, not available; HF, heart failure; LVEF: left ventricular heart failure.

* Standard therapy used were in accordance with recommended clinical guidelines for acute heart failure (titrated diuretic treatment).

† Data is expressed as mean ± standard deviation (SD) or median (IQR).

### Risk of bias in included studies

Of the 7 studies, 3 studies were found to have some concerns of bias (Charaya et al., Thiele et al., Gilani et al.) as a result of bias due to deviations from the intended intervention, measurement of outcomes, during the randomization process, and in the selection of the reported results, while 4 studies were at low risk (Supplementary Figure 1). The key sources of bias included a lack of allocation concealment and the absence of a prespecified analysis plan, which may have led to selection bias and post-randomization imbalances. Furthermore, the failure to implement an intention-to-treat approach in these studies raises concerns about potential attrition bias, which could lead to an overestimation of treatment effects.

## Results of the meta-analyses

### Primary outcomes

#### All-cause mortality

**Figure 2:**
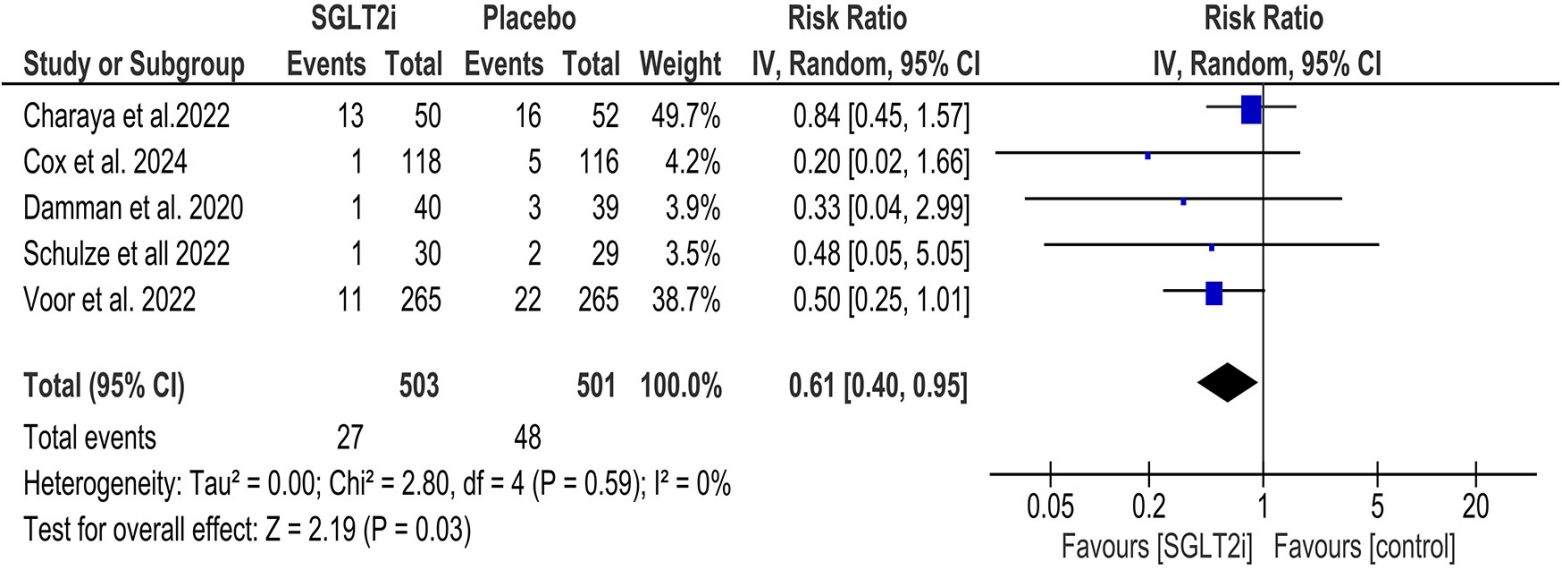
Forest plot of All-cause mortality.

SGLT2 inhibitors significantly decreased all-cause mortality (RR = 0.61, 95% CI = 0.40, 0.95; Figure 2), and statistical heterogeneity was found to be minimal (*I*^2^ = 0%). Subgroup analysis was performed based on the type of SGLT2 inhibitor (empagliflozin and dapagliflozin), and it was found that there was no significant statistical difference between the two subgroups (*P* = 0.77; Supplementary Figure 2). Similarly, subgroup analysis of all-cause mortality based on the duration of follow-up yielded no significant difference between the subgroups (*P* = 0.60; Supplementary Figure 3).

#### Readmission for HF

**Figure 3:**
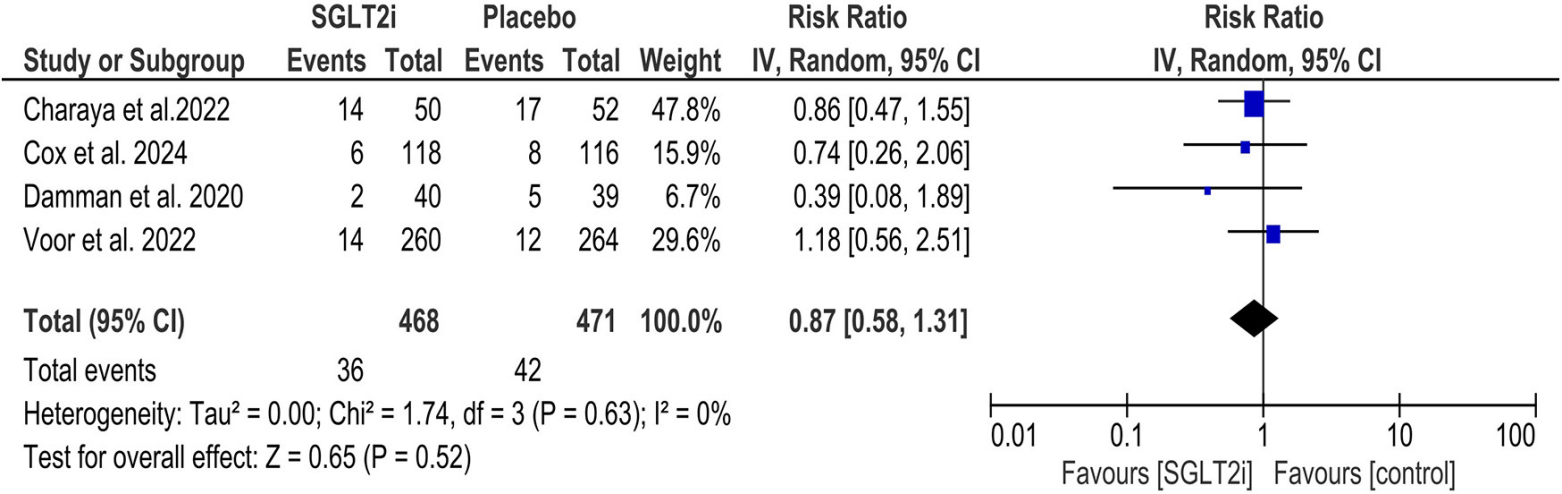
Forest plot of readmission for HF.

SGLT2 inhibitors did not reduce the risk of readmission due to HF (RR = 0.87, 95% CI = 0.58, 1.31; Figure 3), and the statistical heterogeneity was minimal (*I*^2^ = 0%). Subgroup analysis was performed based on the type of SGLT2 inhibitor (empagliflozin and dapagliflozin), and no statistically significant difference was found between the two subgroups (*P* = 0.96; Supplementary Figure 4).

### Secondary outcomes

#### Cardiovascular mortality

SGLT2 inhibitors were not superior to the placebo in reducing cardiovascular mortality in patients with AHF (RR = 0.67, 95% CI = 0.45, 1.00; Supplementary Figure 5) with minimal statistical heterogeneity (*I*^2^ = 0%).

##### AKI

There was no statistically significant difference found in the incidence of AKI between the two groups (RR = 1.46, 95% CI = 0.50, 4.24; Supplementary Figure 6). The statistical heterogeneity was found to be minimal (*I*^2^ = 0%).

#### Hypoglycemia

There was no statistically significant difference between the two groups in terms of hypoglycemic events (RR = 0.90, 95% CI = 0.42, 1.94; Supplementary Figure 7) with minimal statistical heterogeneity (*I*^2^ = 0%).

#### Worsening Hf

SGLT2 inhibitors have been found to significantly reduce the incidence of worsening HF compared to placebo (RR = 0.59, 95%CI = 0.36, 0.97; Supplementary Figure 8). The interstudy heterogeneity was minimal (*I*^2^ = 0%).

##### UTI

There was no statistically significant difference between the two subgroups regarding the incidence of UTIs (RR = 0.59, 95% CI = 0.31, 1.15; Supplementary Figure 9). The statistical heterogeneity was estimated to be minimal (*I*^2^ = 0%).

#### Hypotension

There was no statistically significant difference between the two subgroups in terms of the development of hypotensive episodes (RR = 0.63, 95% CI = 0.29, 1.97; Supplementary Figure 10), with minimal statistical heterogeneity (*I*^2^ = 0%).

#### Diuretic efficiency

No significant difference was found between the two groups in diuretic efficiency (MD −0.29, 95% CI = −0.81, 0.23; Supplementary Figure 1^1^). The statistical heterogeneity was found to be high (*I*^2^ = 65%).

##### GFR

SGLT2 inhibitors were associated with significantly reduced GFR as compared to the control group (MD: 1.05, 95% CI = 0.68, 1.43; Supplementary Figure 1^2^) with minimal statistical heterogeneity (*I*^2^ = 0%).

#### KCCQ-TSS score improvement

SGLT2 inhibitors were found to significantly improve the KCCQ-TSS score compared to the control group (MD: −3.82, 95% CI = −7.51, −0.13; Supplementary Figure 1^3^). The heterogeneity was estimated to be minimal (*I*^2^ = 0%).

## Discussion

In recent years, SGLT2 inhibitors have emerged as potential treatment options for patients with AHF, demonstrating notable efficacy in the management of this complex condition. Several systematic reviews and meta-analyses examining the efficacy of SGLT2 inhibitors in patients with AHF have provided significant insights into their clinical impact.

Our meta-analysis revealed that SGLT2 inhibitors provided a mortality benefit and reduced the rate of worsening HF. However, there was no significant reduction in cardiovascular mortality or HF readmission rates associated with their use. We also did not observe any significant risk of adverse events, aside from a reduction in GFR compared with the control group. Moreover, SGLT2 inhibitors significantly improved the KCCQ-TSS score in chronic HF populations, and recent data from the EMPULSE trial suggest similar improvements in quality of life among patients hospitalized with AHF. Our findings also indicate a substantial reduction in all-cause mortality by approximately 39%, highlighting the potential role of these agents as therapeutic options in the management of HF. This mortality benefit aligns with the existing literature on SGLT2 inhibitors, reinforcing their role as a treatment option for chronic HF management and in the acute setting.

The efficacy of SGLT2 inhibitors was initially evaluated in the EMPA-REG OUTCOME trial. In this trial, patients hospitalized for HF who received SGLT2 inhibitors experienced a significantly reduced risk of rehospitalization or death within the first three months following an initial HF event (HFE) (29). In another trial, the EMPULSE trial (26) evaluated patients over the first 90 days after hospital admission, a period often considered the vulnerable phase of HF. This trial included patients with no prior history of HF (acute *de novo*) who had not yet been treated for HF. This demonstrated that adding empagliflozin to standard therapy was well tolerated and produced clinical benefits similar to those seen in patients with chronic decompensated HF. Clinical benefits in the EMPULSE trial included a composite of death, number of HFEs [including hospitalizations for HF (HHFs), urgent HF visits, and unplanned outpatient visits], time to first HFE, and change from baseline in the KCCQ-CSS after 90 days of treatment. An extended pilot study of this trial, called EMPA-RESPONSE-AHF, also suggested a clinical benefit of empagliflozin in patients hospitalized for AHF (28). These results indicate that empagliflozin is highly regarded as an effective treatment for patients hospitalized with both *de novo* and decompensated AHF.

Several large-scale drug trials for hospitalized patients with AHF have failed to demonstrate substantial benefits, potentially due to the short duration of therapy (24–48 h) or the lack of continuation post-discharge (30–34). While the PIONEER-HF trial, with a design similar to EMPULSE, focused on NT-proBNP levels rather than clinical outcomes (35), subsequent trials such as EMPULSE and TRANSITION have provided evidence supporting the safety of initiating chronic HF therapies, such as SGLT2 inhibitors and sacubitril-valsartan, respectively, during the index hospitalization (pre-discharge) (36, 37). Nonetheless, questions remain regarding the mechanisms behind the lack significant reduction in re-hospitalization rates seen with SGLT2 inhibitors in meta-analyses for AHF, especially when compared to their pronounced impact on reducing re-hospitalizations in chronic HF populations. Our results also did not show a significant reduction in HF readmission with SGLT2 inhibitors. This raises critical questions regarding the long-term management of AHF. Although these agents reduce all-cause mortality and improve symptoms, their limited effect on preventing readmissions suggests that more research is needed to understand their role in preventing HF progression vs. stabilizing patients' post-acute events. A new trial is currently evaluating the effects of in-hospital dapagliflozin initiation in patients hospitalized with AHF post-stabilization between days 1 and 14 (38).

A review by Salah et al., which specifically addressed the initiation of SGLT2 inhibitors in patients hospitalized for AHF, demonstrated a significant reduction in the risk of rehospitalization for HF and improvement in patient-reported outcomes without an increase in adverse effects such as AKI, hypotension, or hypoglycemia. Similarly, a review by Kumar et al. (39) reported a significant reduction in cardiovascular death or HHF associated with SGLT2 inhibitor use alongside a decrease in HF symptoms and comparable rates of adverse events. Kumar et al. also reported a reduction in all-cause mortality associated with SGLT2 inhibitor use, whereas Salah et al. did not find a statistically significant effect. Moreover, review studies by Roy et al. (40) and Ahmad et al. (41) support the efficacy of SGLT2 inhibitors in reducing HHF and cardiovascular death across a broader patient population, not limited to AHF. Huang et al. (42) also demonstrated the efficacy of SGLT2 Inhibitors in patients with HFpEF. In support of this, another study showed that SGLT2 inhibitors are effective irrespective of EF status or the presence of diabetes mellitus (43). This suggests that the benefits of SGLT2 inhibitors are not confined to a specific type of HF. Therefore, SGLT2 inhibitors should be considered a valuable addition to the treatment regimen for patients with HF, including those with AHF. However, these meta-analyses included the SOLOIST-HF trial (44), which included patients who continued to use SGLT2 inhibitors even after discharge. In our meta-analysis, we analyzed trials that included patients treated within five days of AHF presentation to preserve homogeneity.

Our study performed a subgroup analysis of the specific SGLT2 inhibitors, empagliflozin and dapagliflozin. Interestingly, this analysis did not reveal any statistically significant differences in the efficacy between the two drugs. This finding suggests a class effect inherent to SGLT2 inhibitors, offering clinicians a level of reassurance regarding the interchangeable use of these agents in practice. The absence of a significant difference can guide treatment decisions, especially in resource-limited settings or when patient preferences must be considered. We also performed a subgroup analysis for all-cause mortality based on the duration of follow-up, but no significant differences were found between the subgroups. Another notable finding in our meta-analysis revealed that SGLT2 inhibitors reduced the worsening of HF compared to placebo, with a relative risk reduction of 41%. This large risk reduction highlights their potential role in symptom management, which is critical for improving the quality of life of patients with HF. There was also a statistically significant improvement in the KCCQ-TSS score. This presents a compelling argument for incorporating SGLT2 inhibitors into the standard care protocols for patients with AHF.

The use of SGLT2 inhibitors is believed to overcome the drawback of diuretic resistance often seen with diuretics alone in patients with AHF. This occurs by counteracting sodium absorption in PCT, thereby exerting a natriuretic effect (45, 46). However, our review did not find any significant improvements in diuretic efficacy, suggesting that other mechanisms may have been involved. Nevertheless, clinicians should be vigilant regarding individual patient responses and potential diuretic-related adverse effects. Inhibition of sodium and glucose reabsorption in the proximal tubule by SGLT2 inhibitors leads to an increased delivery of sodium to the macula densa. This triggers tubuloglomerular feedback, causing afferent arteriolar constriction, and thus, a reduction in eGFR. This decline is seen early within 2–4 weeks of initiation while showing some recovery by week 12 (47). However, this decrease in GFR appears to be transient, with no bearing on its protective effect on cardiovascular outcomes (48).

The safety profile of SGLT2 inhibitors appears robust, with no statistically significant differences noted between the SGLT2 inhibitor and control groups concerning AKI, hypoglycemia, UTIs, or hypotension. This suggests that when prescribed judiciously, SGLT2 inhibitors can be considered safe adjuncts in the treatment of AHF without imposing substantial risks commonly associated with HF pharmacotherapy.

Our study employed a comprehensive search strategy across multiple databases to capture all relevant RCTs. Additionally, we included only those studies that assessed the impact of SGLT2 inhibitors in patients within the first five days of AHF onset, enhancing the consistency of our findings.

However, several limitations of this meta-analysis should be acknowledged. The individual trials varied in key aspects, such as the type of control group, time from hospital admission to randomization, treatment duration, follow-up period, and HF severity, all of which contributed to heterogeneity. Importantly, not all the outcomes were evaluated in every study. For example, mortality was assessed in only five of the seven included trials, and safety outcomes such as adverse events were reported inconsistently across studies. This variability in reported outcomes limits the ability to draw definitive conclusions about certain endpoints and may introduce potential reporting bias. While statistical heterogeneity was minimal in most of our meta-analyses, these variations in study design and outcome definitions should be considered when interpreting our findings. Additionally, our study did not perform a subgroup analysis based on the type of AHF (recurrent vs. *de novo*) or EF (HFrEF vs. HFpEF), which may have provided further clinical insights. Certain safety outcomes were evaluated in only a few trials, limiting our ability to detect significant effects. Furthermore, some trials excluded patients with specific conditions, such as ESRD and ACS, making our findings less applicable to these populations. Finally, we relied on published summary data rather than individual patient-level data, which could have provided more precise patient-level insights, better identification of exposures and outcomes, and greater adjustment for confounders to reduce heterogeneity (49).

Given the limitations identified in this meta-analysis, future research should address the variability between trials. Standardizing aspects such as control group selection, treatment duration, follow-up periods, and the time from hospital admission to randomization will help reduce heterogeneity and improve the comparability of the results. Additionally, future studies should focus on different types of AHF (chronic decompensated vs. *de novo*) to better understand differential treatment effects. More robust investigations into the safety outcomes of SGLT2 inhibitors, particularly in underrepresented populations, such as those with ESRD or ACS, are needed.

In conclusion, the findings of this systematic review and meta-analysis underscore the overall clinical benefits and favorable profile of SGLT2 inhibitors in patients with AHF. However, their limited impact on readmission rates indicates the need for a nuanced approach to HF management that incorporates both pharmacological and non-pharmacological strategies. Further research is warranted to better characterize patient populations that derive the greatest benefit and to refine treatment strategies for optimal clinical outcomes.

## Data Availability

The original contributions presented in the study are included in the article/Supplementary Material, further inquiries can be directed to the corresponding author.
