# Supplementary material for: Efficacy and safety of SGLT2 inhibitors in acute heart failure: a systematic review and meta-analysis of randomized controlled trials
Source: Front Cardiovasc Med. 2025 May 1;12:1543153. doi: 10.3389/fcvm.2025.1543153 (PMC12078209; doi:10.3389/fcvm.2025.1543153)
Supplement: Supplementary file 1 [file Datasheet1.docx]

# Supplementary Material

# Efficacy and safety of SGLT2 inhibitors in acute heart failure: a systematic review and meta-analysis of randomized controlled trials

This supplemental material has been provided by the authors to give readers additional information about their work.

**Supplementary Figure 1. Risk of bias assessment of RCTs**
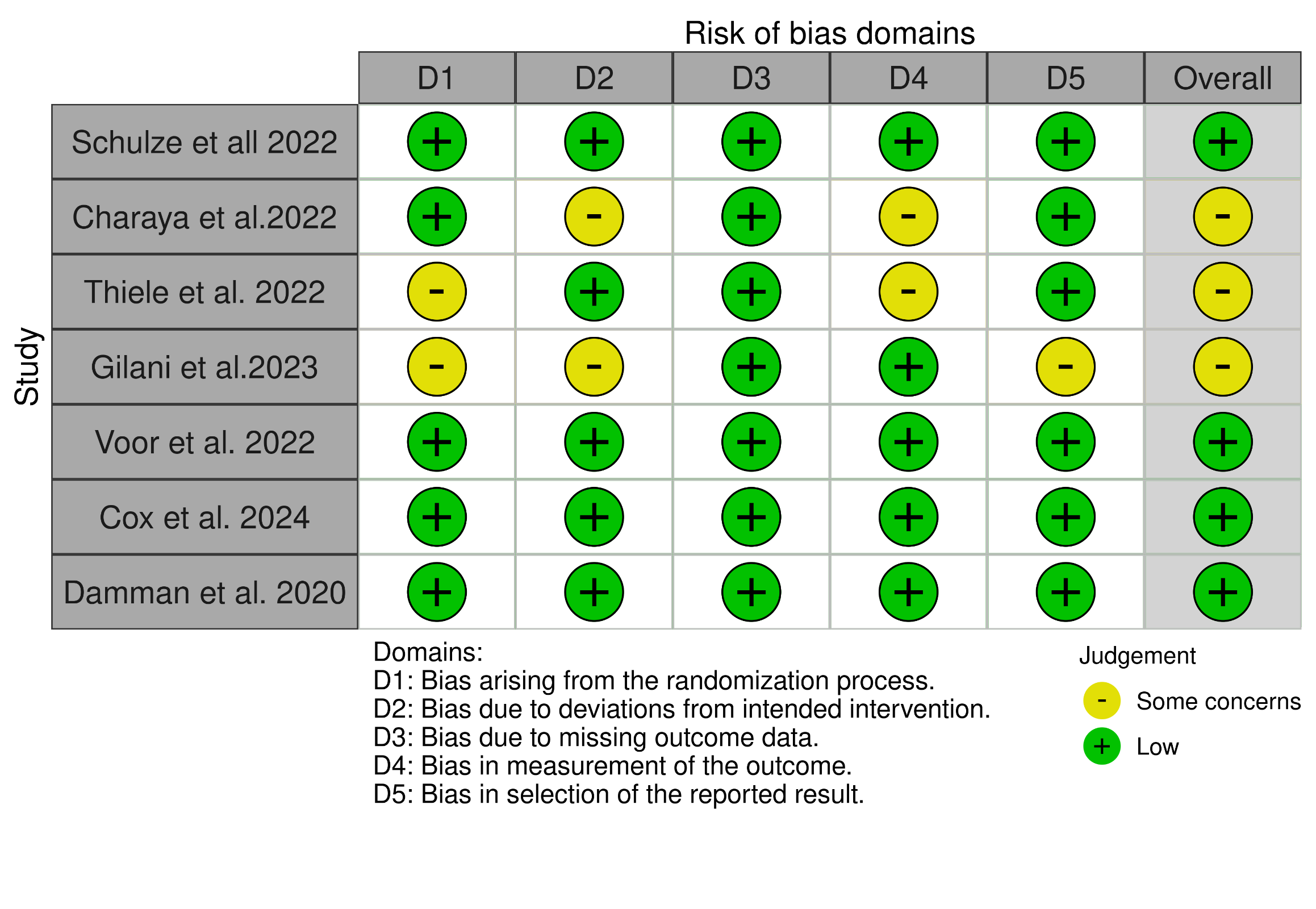


**Supplementary Figure 2. Subgroup analysis of All-cause mortality based on type of SGLT2 inhibitor**

**
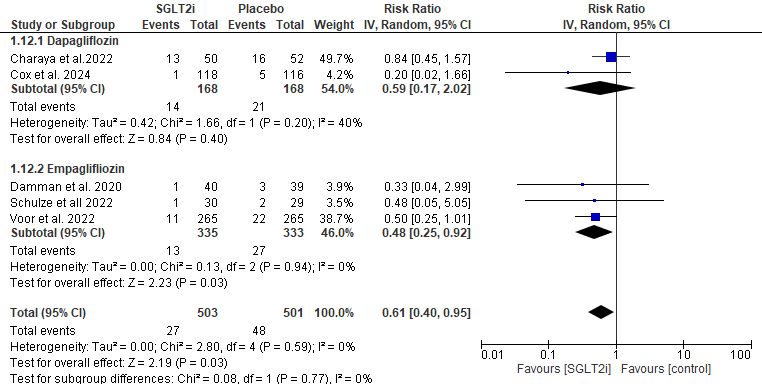
**

**Supplementary Figure 3. Subgroup analysis of All-cause mortality based on the duration of follow-up**

**
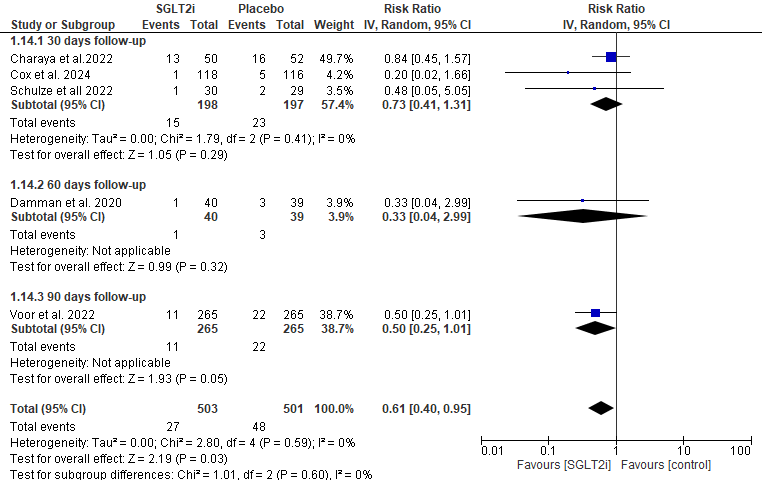
**

**Supplementary Figure 4. Subgroup analysis of readmission for HF based on type of SGLT2 inhibitor**

**
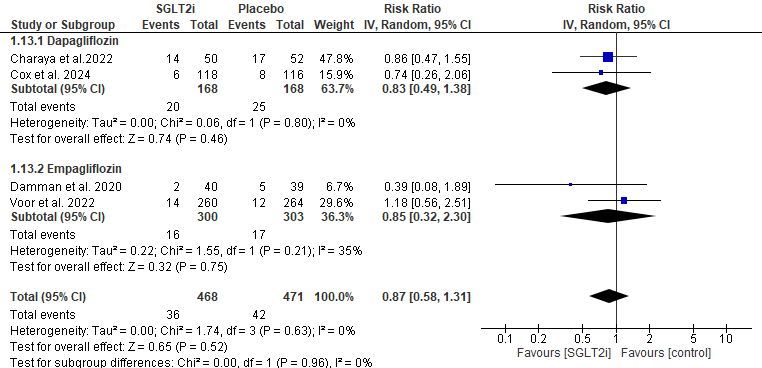
**

**Supplementary Figure 5. Forest plot of CV mortality**

**
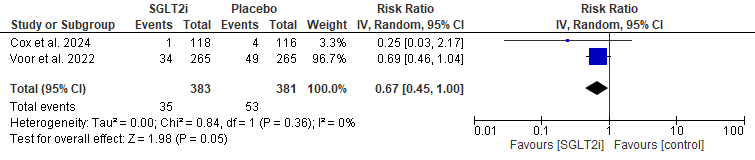
**

**Supplementary Figure 6. Forest plot of AKI**

**
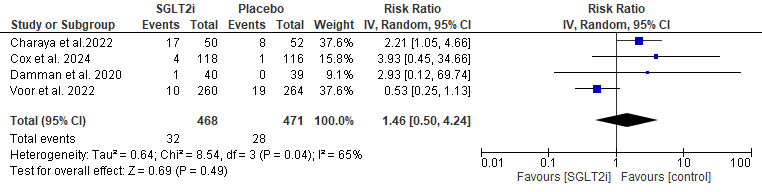
**

**Supplementary Figure 7. Forest plot of Hypoglycemia**

**
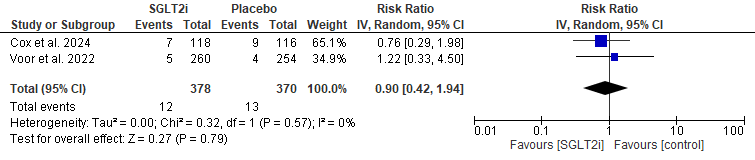
**

**Supplementary Figure 8. Forest plot of worsening HF**

**
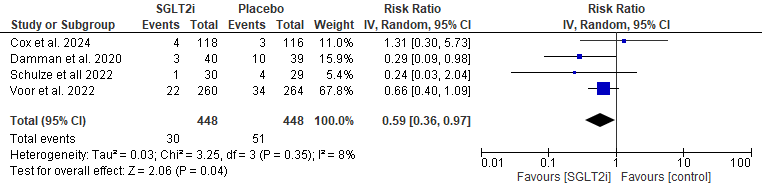
**

**Supplementary Figure 9. Forest plot of UTI**

**
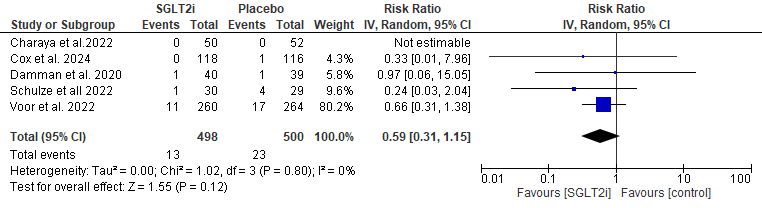
**

**Supplementary Figure 10. Forest plot of Hypotension**

**
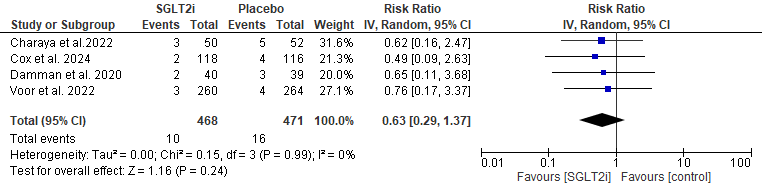
**

**Supplementary Figure 11. Forest plot of Diuretic efficiency**

**
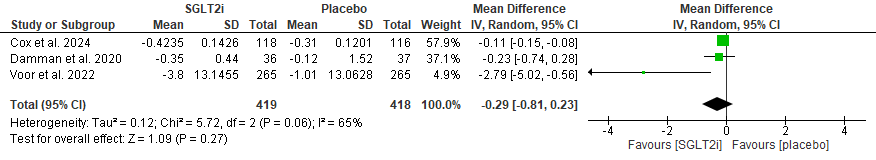
**

**Supplementary Figure 12. Forest plot of GFR**

**
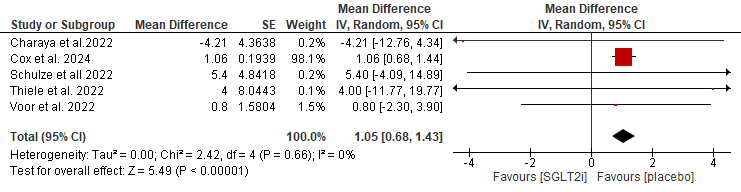
**

**Supplementary Figure 13. Forest plot of KCCQ-TSS improvement**

**
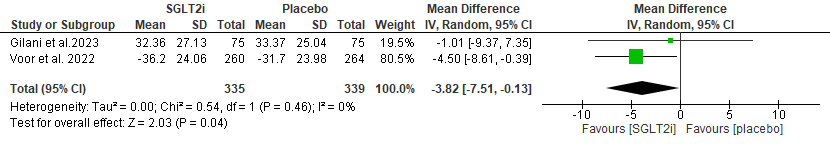
**
